# Malaria rapid diagnostic tests in community pharmacies in Rwanda: availability, knowledge of community pharmacists, advantages, and disadvantages of licensing their use

**DOI:** 10.1186/s41256-023-00324-z

**Published:** 2023-09-12

**Authors:** Amon Nsengimana, Joyce Isimbi, Theogene Uwizeyimana, Emmanuel Biracyaza, Jean Claude Hategekimana, Charles Uwambajimana, Olivia Gwira, Vedaste Kagisha, Domina Asingizwe, Ahmed Adedeji, Jean Baptiste Nyandwi

**Affiliations:** 1 USAID Global Health Supply Chain Program-Procurement and Supply Management, Kigali, Rwanda; 2 Department of Pharmacy, School of Medicine and Pharmacy, University of Rwanda, Kigali, Rwanda; 3 University of Global Health Equity, Kigali, Rwanda; 4 School of Rehabilitation, Faculty of Medicine, University of Montreal, Montréal, QC Canada; 5 Department of Physiotherapy, School of Health Sciences, University of Rwanda, Kigali, Rwanda; 6 East African Community Regional Center of Excellence for Vaccines, Immunization and Health Supply Chain Management, Kigali, Rwanda

**Keywords:** Availability, Knowledge, Malaria rapid diagnostic test, Community pharmacists

## Abstract

**Background:**

Presumptive treatment of malaria is often practiced in community pharmacies across sub-Saharan Africa (SSA).To address this issue, the World Health Organization (WHO) recommends that malaria Rapid Diagnostic Tests (m-RDTs) be used in these settings, as they are used in the public sector. However, their use remains unlicensed in the community pharmacies in Rwanda. This can lessen their availability and foster presumptive treatment. Therefore, this study investigated the availability of m-RDTs, knowledge of community pharmacists on the use of m-RDTs, and explored Pharmacists’ perceptions of the advantages and disadvantages of licensing the use of m-RDTs in community pharmacies.

**Methods:**

This was a cross-sectional study among 200 licensed community pharmacists who were purposefully sampled nationwide from 11th February to 12th April 2022. Data was collected using an online data collection instrument composed of open-ended and closed-ended questions. Statistical analyses were performed using the Statistical Package for the Social Sciences (SPSS) version 25.0. The chi-square test was used to evaluate the association between the availability of m-RDTs and independent variables of interest. Content analysis was used for qualitative data.

**Results:**

Although 59% were consulted by clients requesting to purchase m-RDTs, only 27% of the participants had m-RDTs in stock, 66.5% had no training on the use of m-RDTs, and 18.5% were not at all familiar with using the m-RDTs. Most of the participants (91.5%) agreed that licensing the use of m-RDTs in community pharmacies could promote the rational use of antimalarials. The chi-square test indicated that being requested to sell m-RDTs (x^2^ = 6.95, p = 0.008), being requested to perform m-RDTs (x^2^ = 5.39, p  = 0.02),familiarity using m-RDTs (x^2^ = 17.24, p = 0.002), availability of a nurse in the Pharmacy (x^2^ = 11.68, p < 0.001), and location of the pharmacy (x^2^ = 9.13, p = 0.048) were all significantly associated with the availability of m-RDTs in the pharmacy.

**Conclusions:**

The availability of m-RDTs remains low in community pharmacies in Rwanda, and less training is provided to community pharmacists regarding the use of m-RDTs. Nevertheless, community pharmacists had positive perceptions of the advantages of licensing the use of m-RDTs. Thus, licensing the use of m-RDTs is believed to be the first step toward promoting the rational use of antimalarial medicines in Rwanda.

## Background

Malaria continues to be a public health problem worldwide [1, 2]. In 2021,the World Health Organization (WHO) reported 619 thousand deaths and 247 million cases of malaria globally [2]. Although malaria is a global public health concern [1], Africa bears the highest burden of the disease compared with other regions [1, 3]. In 2021, Africa accounted for about 95% of all reported cases of malaria worldwide, with only four African countries—Nigeria, the Democratic Republic of Congo, the United Republic of Tanzania, and Niger—accounting for over half of all malaria deaths globally [2]. Like most other African countries, malaria is one of Rwanda’s foremost public health problems taking the greatest toll on children under five years and pregnant women [4]. However, the entire Rwandan population is also at risk for contracting malaria, with 19 out of its 30 districts prone to epidemics, and malaria being endemic in 11 districts [5]. In 2021, there were 1,163,670 cases of malaria and 60 related deaths in Rwanda [2].

Globally, early diagnosis and prompt treatment of malaria with antimalarial medicines remain the cornerstone of malaria control [6]. In sub-Saharan Africa (SSA), the primary source of antimalarial medicines is private health providers, including community pharmacies [7, 8]. A study in this region reported that community pharmacies account for between 33% and 95% of the market share of antimalarial medicines sales volume [9]. In some countries like Nigeria, community pharmacies are typically the first point of contact in the healthcare system for the management of minor health problems, including malaria [8]. Similarly, in Kenya, the private for-profit market sector accounts for 50–80% of the market share for malaria treatment, and 34–42% of children rely on antimalarials purchased from retail drug outlets for their first or only treatment [10]. Moreover, in Uganda, up to 80% of patients seek treatment from the private sector including community pharmacies [11]. These studies show that community pharmacies play a vital role in malaria treatment in SSA. This is because public health facilities are often distantly located, have limited staff, long waiting times, limited hours, and frequently run out of essential medicines [12]. Thus, many people opt to visit community pharmacies for treatment despite the relatively low cost of care in public health facilities [12].

Although community pharmacies are the preferred choice for malaria treatment among SSA patients, they usually do not provide malaria diagnostic tests [13, 14]. This contributes to antimalarial drug resistance, which poses a serious threat to public health [15]. To address this issue, the WHO has recommended the use of malaria rapid diagnostic tests (m-RDTs) in the private sector, as in the public sector [6]. Since then, most malaria-endemic countries have introduced the use of m-RDTs at various levels of community pharmacies, and pharmacists have demonstrated willingness to implement this policy [14]. However, this policy has not yet been implemented in the community pharmacies in Rwanda. Therefore, because of the lack of implementation of relevant policies, m-RDTs can only be purchased in these settings, but their use remains unlicensed. Even ,there is no collaboration between the government to supply m-RDTs through community pharmacies. Consequently, community pharmacies rely on private wholesale pharmacies that import m-RDTs from international manufacturers or distributors.

The lack of implementation of the WHO’s recommendation to expand access to malaria diagnosis through community pharmacies in Rwanda raises concerns, especially given the country’s report on artemisinin partial resistance in 2020 [16]. Rwanda’s report on artemisinin partial resistance coincided with the identification of community pharmacies as a significant source of self-medication [17].Although the causes and development of antimalarial drug resistance are complex, self-medication with antimalarials is among the main contributing factors [18]. Regrettably, little is known about the availability of m-RDTs in community pharmacies, the knowledge of community pharmacists regarding the use of m-RDTs, and community pharmacists’ perceptions of the need for licensing the use of m-RDTs in these settings. To attain universal access to malaria diagnosis and treatment, scholars have reported that it requires closing the testing availability gap between the public and private sectors [19]. Hence, this study aimed to [1] assess the availability of m-RDTs in community pharmacies in Rwanda, [2] determine the knowledge of community pharmacists regarding the use of m-RDTs, and [3] explore community pharmacists’ perceptions of the advantages and disadvantages of licensing the use of m-RDTs in community pharmacies.

Analyzing the availability of m-RDTs in community pharmacies can inform the development of new policies to reduce possible presumptive treatment and improve access to malaria diagnosis in community pharmacies, where accurate diagnosis and appropriate treatment for malaria are essential to prevent the development and spread of antimalarial drug resistance. Moreover, identifying any knowledge gaps in the use of m-RDTs will also inform the development of targeted training programs to improve community pharmacists’skills and increase their confidence in using m-RDTs. Such programs will be especially valuable when new policies that require the use of m-RDTs before dispensing antimalarial medicines are introduced. Finally, understanding the views and perceptions of community pharmacists on the advantages and disadvantages of licensing the use of m-RDTs is crucial, as they are considered key healthcare professionals in addressing the issue of antimalarial drug resistance.

## Methods

### Study design and setting

A cross-sectional study was conducted from 11th February to 12th April 2022 among 200 licensed community Pharmacists in Rwanda, using both quantitative and qualitative approaches.

The pharmaceutical workforce in Rwanda consists of pharmacists and pharmacy Technicians [20]. The 9th edition of the pharmacy professionals’ register counts 1138 Pharmacists and 12 pharmacy technicians working in diverse sectors, with most pharmacists working in community pharmacies [21]. Pharmacy is a regulated profession overseen by Rwanda Food and Drugs Authority (Rwanda FDA) and before practicing, Pharmacists must be licensed by the Rwanda National Pharmacy Council (NPC). A community pharmacy in Rwanda is commonly referred to as a private retail pharmacy, while a community pharmacist is defined as a registered and licensed pharmacist with a bachelor’s degree in pharmacy or Doctor of Pharmacy (Pharm D) working in a private retail pharmacy [21]. In this study, community pharmacies and community pharmacists were used interchangeably to refer to private retail pharmacies and pharmacists, respectively. A list of licensed community pharmacies showed that there were 612 licensed retail Pharmacies during the study period [22].

### Sample size and sampling techniques

To determine the sample size, the Yamane formula was used [23] $\text{n}\text{Y}=\frac{\text{N}}{1+\text{N}{\text{e}}^{2}}$ Where “N” stands for population size, “e” for alpha level (e = 0.05) at the confidence interval of 95%, and nY is the sample size. The population size used was a list of licensed human retail Pharmacies in Rwanda as of 2021, the latest version available during the study period. Therefore, $nY=\frac{612}{1+612(0.0{5)}^{2}}=$ 242. The resulting sample size was recruited nationwide through a purposive sampling method, which involved the recruitment of a single pharmacist to represent each community pharmacy. Unlicensed pharmacists and those who declined to participate voluntarily were excluded. Out of 242 community Pharmacists to whom the data collection instrument was shared, 200 responded, resulting in a response rate of 83%.

### Data collection instrument

The data collection instrument was self-administered and included both open-ended and close-ended questions. It consisted of 40 items grouped into five sections: (A): Characteristics of community Pharmacists and Pharmacies, (B): Availability and request of malaria rapid diagnostic tests in community pharmacies, (C): Knowledge of Community Pharmacists on using m-RDTs, (D): Perceptions of community Pharmacists towards the advantages and disadvantages of using m-RDTs in community pharmacies. This section used a two-Likert scale (1 = Disagree and 2 = Agree) and had 10 statements for both advantages and disadvantages, (E).Views of community Pharmacists on what can be done to improve their contribution to malaria management. In this section, to gather opinions on ways to enhance their contribution to malaria management, an open-ended question was utilized.

The data collection instrument was set up limited to a single submission. Setting up the data collection instrument to allow for only a single submission ensured that each participant completes the survey only once, which helped to reduce the risk of response bias. Moreover, designing a data collection instrument using skip logic has also been a useful way to improve the efficiency and accuracy of data collection. Skip logic further allowed to tailor the survey questions to each participant based on their previous responses. This helped to ensure that participants only respond to questions that apply to them, which reduced survey fatigue and increased the accuracy of the data collected.

### Validity and reliability of the instrument

A pilot study involving 24 community pharmacists was carried out to ensure the validity and dependability of the data collection instrument. The sub-component with Cronbach’s Alpha greater than 0.6 was deemed consistent. Two untrustworthy questions were critically changed to make them more understandable to the study participants. The data collection instrument demonstrated generally good internal consistency, Cronbach’s Alpha (α) = 0.89.

### Data collection procedure

Each community pharmacy was represented by a single recruited Pharmacist. The data collection started from 11th February to 12th April 2022. All community pharmacists of any age were included. Data collectors who were trained in research ethics approached only licensed community pharmacists who accepted to participate in this study. To minimize selection bias, community Pharmacists from each district in the country were approached, and thus each district in Rwanda was represented.

### Data analysis

Data cleaning for the quantitative data collected was carried out using Microsoft Excel. The cleaned data was thereafter imported into the Statistical Package for Social Sciences (SPSS) version 25.0 for Windows and then double-checked for accuracy. Quantitative data were presented as frequencies, percentages, means, and standard deviations for descriptive statistics. To perform analytical analyses, a chi-square test was used to assess the association between independent variables of interest and the availability of m-RDTs in community pharmacies. A p-value of less than 0.05 was regarded as statistically significant. A two-point Likert scale was used in section D to assess the perceptions of community pharmacists on the advantages and disadvantages of m-RDTs in community pharmacies, with responses ranging from agree to disagree. The responses of agree and disagree on each statement were calculated as the mean scores and standard deviation. Scores above the midpoint of 1.5 were taken as positive attitudes towards advantages, while scores below 1.5 were considered as negative attitudes towards disadvantages, indicating the stated disadvantages. The final section (Section E) was qualitative and was analyzed using content analysis. Texts made by Community Pharmacists were read carefully, thoroughly and classified. The content that fell under identified topics was then placed under their respective themes.

## Results

### Characteristics of community pharmacists

Of 242 sampled community pharmacists contacted to participate, only 200 participated yielding a response rate of 83%. Our results showed that 58% (n = 116) of the participants were single, and the majority, 90.5% (n = 181) were between the age of 25 and 35, with an overall average age of 31 (SD = 5.2). Of the 200 participants, 74% of them (n = 148) were male, while 26% (n = 52) were female. The majority, 90.5% (n = 181) had a bachelor’s degree, and a few, 9.5% (n = 19) had a master’s. Participants enrolled in this study graduated between 1987 and 2021, with the majority, 74% (n = 148) graduating between 2016 and 2021. The majority of community Pharmacists, 71% (n = 142) had less than six years of experience working in community pharmacy (Table 1).

**Table 1:** Characteristics of community pharmacists (n = 200)

Variables	Frequency (n)	Percent (%)
Sex		
Male	148	74
Female	52	26
Age		
25–35 years	181	90.5
36–45 years	17	8.5
46 and above	2	1
Education		
Bachelor’s degree	181	90.5
Master’s degree	19	9.5
Marital status		
Single	116	58
Married	84	42
University of graduation		
University of Rwanda	164	82
Mount Kenya University	25	12.5
Foreign university	11	5.5
Graduation year		
< 2005	2	1
2005–2010	6	3
2011–2015	44	22
2016–2021	148	74
Experience working in a community pharmacy		
1–5 years	142	71
6–10 years	50	25
11–15 years	8	4

### Characteristics of community pharmacies

More than half of the participants, 56% (n = 112) were employed in Kigali city, the capital city of Rwanda. Among the participants, 79%(n = 158) were employees in community pharmacies, while only 21%(n = 42) were pharmacy owners. A large proportion, 91%(n = 182) of the participants reported having nurses as other healthcare providers available in their community pharmacies (Table 2).

**Table 2:** Characteristics of community pharmacies (n = 200)

Variables	Frequency (n)	Percent (%)
Location of pharmacy		
Kigali city	112	56
Southern Province	24	12
Northern Province	24	12
Western Province	18	9
Eastern Province	22	11
Ownership of pharmacy		
Owner	42	21
Employee	158	79
Availability of a Nurse in the pharmacy		
Yes	182	91
No	18	9
Number of Nurses available in the pharmacy		
1 Nurse	61	30.5
2–4 Nurses	131	65.5
5–6 Nurses	8	4

### Availability and request of of m-RDTs in community pharmacies in Rwanda

Table 3 shows that 73% (n = 146) of community Pharmacists did not have m-RDTs in stock, while only 27% (n = 54) had them in stock. Despite this, 69% (n = 138) of community Pharmacists were consulted by clients requesting to purchase m-RDTs. Of those consulted, 72% (n = 99) were requested to sell m-RDTs more than once a week.with at least 59% (n = 118) of the clients further requesting Pharmacists to perform m-RDT from the community pharmacy settings.

**Table 3:** Availability and request of malaria rapid Diagnostic test (n = 200)

Variables	Categories	Frequency (n)	Percent (%)
Availability of m-RDT in the stock during the study period	Yes	54	27
	No	146	73
Requested to sell m-RDT in last week	Yes	138	69
	No	62	31
Frequency of requests to sell m-RDT in a week	1 time	39	28
	2–3 times	48	35
	4–5 times	11	8
	More than 5 times	40	29
Requested to perform m-RDT prior to dispensing antimalarial medicines	Yes	118	59
	No	82	41

*N/A* not applicable, *N* Frequency, *m-RDT* malaria rapid diagnostic test, % percentage

### Knowledge and familiarity of community pharmacists on using m-RDTs

Table 4 shows the knowledge of community pharmacists on the use of m-RDTs. Of the 200 participants, 18.5% (n = 37) were not at all familiar with the use of m-RDTs, while 66.5% (n = 133) reported that they did not receive any training on the use of m-RDTs. However, 33.5% (n = 67) of the study participants had received training on the use of m-RDTs. The mean score of familiarity stood at 2.23 (with a standard deviation of 1.42), placing it below the midpoint of 3 on the five-level Likert scale. This suggests a lower level of familiarity.

**Table 4:** Knowledge and familiarity of Community Pharmacists on using m-RDTs (n = 200)

Variables	Categories	Frequency(n)	Percent(%)
Familiarity with the use of m-RDTs	Not at all familiar	37	18.5
	Slightly familiar	30	15
	Somewhat familiar	27	13.5
	Moderately familiar	63	31.5
	Extremely familiar	43	21.5
Trained on using m-RDTs	Yes	67	33.5
	No	133	66.5
Number of training received	1 training	27	40.3
	2–4 training	29	43.3
	5–8 training	11	16.4

n: Frequency, %: percentage

### Association between participants’ characteristics and availability of m-RDTs

The Chi-square analysis conducted revealed several factors that were significantly associated with the availability of m-RDTs. Specifically,the Chi-square results indicated that a significantly higher proportion of community pharmacists working in Kigali City (59.3%) stocked m-RDTs compared to those in the provinces, (x^2^ = 9.13, p = 0.048). Additionally, community pharmacists who worked with nurses in their pharmacies (79.6%) had significantly higher availability of m-RDTs in their stock, (x^2^ = 11.68, p < 0.001). Furthermore, community pharmacists who were requested to sell m-RDTs (74.1%) had a significantly higher stock of m-RDTs than those who were not, (x^2^ = 6.95, p = 0.008). Similarly, community pharmacists who were moderately familiar with using m-RDTs (38.9%) significantly had m-RDTs in stock, (x^2^ = 17.24, p = 0.002) (Table 5).

**Table 5:** Association between participants’ characteristics and availability of m-RDTs

Variables	Availability of m-RDTs				X^2^	p-value
	No		Yes			
Sex	n	%	n	%		
Male	112	76.7	36	66.7	2.07	0.15
Female	34	23.3	18	33.3		
Age						
25–35 years	129	88.4	52	96.3	3.02	0.21
36–45 years	15	10.3	2	3.7		
46 and above	2	1.45	0	0		
Education						
Bachelor’s degree	131	89.7	50	92.6	0.38	0.53
Master’s degree	15	10.3	4	7.4		
Marital status						
Single	81	55.5	35	64.8	1.41	0.23
Married	65	44.5	19	35.2		
University of graduation						
University of Rwanda	121	82.9	43	79.6	9.08	0.01*
Mount Kenya University	21	14.4	4	7.4		
Foreign university	4	2.7	7	13		
Graduation year						
< 2005	2	1.4	0	0	5.95	0.11
2005–2010	6	4.1	0	0		
2011–2015	36	24.7	8	14.8		
2016–2021	102	69.9	46	85.2		
Experience in community pharmacy						
1–5 years	99	67.8	43	79.6	2.87	0.23
6–10 years	40	27.4	10	18.5		
11–15 years	7	4.8	1	1.9		
Location of pharmacy						
Kigali city	80	54.8	32	59.3	9.13	0.04*
Southern Province	20	13.7	4	7.4		
Northern Province	22	15.1	2	3.7		
Western Province	11	7.5	7	13		
Eastern Province	13	8.9	9	16.7		
Ownership of pharmacy						
Owner	31	21.2	11	20.4	0.02	0.89
Employee	115	78.8	43	79.6		
Availability of a Nurse in the pharmacy						
No	7	4.8	11	20.4	11.68	< 0.001***
Yes	139	95.2	43	79.6		
Requested to sell m-RDTs						
No	68	46.6	14	25.9	6.95	0.008**
Yes	78	53.4	40	74.1		
Familiarity using m-RDTs					17.24	0.002**
Not at all familiar	34	23.3	3	5.6		
Slightly familiar	27	18.5	3	5.6		
Somewhat familiar	16	11	11	20.4		
Moderately familiar	42	28.8	21	38.9		
Extremely familiar	27	18.5	16	29.6		
Trained about the use of m-RDTs					5.19	0.42
No	99	67.8	34	63		
Yes	47	32.2	20	37		

${x}^{2}$: Pearson Chi-square value *: Statistical significance at p < 0.05; **: Statistical significance level at p < 0.01, ***: statistical significance at p < 0.001

### Perceived advantages of licensing use m-RDT in community pharmacies

Table 6 shows the level of acceptance of the advantages of licensing m-RDTs in community pharmacies. Of the participants, 91.5% (n = 183) were in agreement that using these tests in community pharmacies could promote the rational use of antimalarial medicines, provide patients with alternative treatment options in the case of a negative test, and decrease the use of malaria medicines for other conditions with similar symptoms. The mean score for the stated advantages ranged from 1.86 (SD = 0.35) to 1.92 (SD = 0.28), indicating a high level of positive acceptance among the participants.

**Table 6:** Perceived advantages of licensing use of m-RDT in community pharmacies

Items	Disagree		Agree		Mean (SD)
Licensing malaria Rapid diagnostic in community Pharmacies can:	n	%	n	%	
Prevent long queues of people at public facilities seeking malaria treatment	27	13.5	173	86.5	1.87 (0.34)
Save people’s time and money by traveling to public health facilities for malaria treatment.	28	14.0	172	86.0	1.86 (0.35)
Prevent the use of antimalarial medicines without malaria confirmation	18	9.0	182	91.0	1.91 (0.29)
Reduce the use of malaria medicines for other malaria-like symptoms	17	8.5	183	91.5	1.91 (0.28)
Increase adherence to the recommended treatment after a test	19	9.5	181	90.5	1.91 (0.29)
Help patients to find alternative treatment in case of a negative test.	17	8.5	183	91.5	1.92 (0.28)
Promote rational use of anti-malaria medicines in community Pharmacies.	17	8.5	183	91.5	1.92 (0.28)
Prevent presumptive treatment of malaria in community Pharmacies	21	10.5	179	89.5	1.90 (0.31)
Detect early malaria, thus early management.	27	13.5	173	86.5	1.87 (0.34)
Prevent antimalarial drug resistance.	23	11.5	177	88.5	1.89 (0.32)

n: Frequency, %: Percentage; SD = Standard deviation

### Perceived disadvantages of licensing use of m-RDT in community pharmacies

Between 52% and 78% of participants disagreed with 8 of the 10 stated disadvantages, considering them to be unlikely disadvantages of using malaria rapid diagnostic tests in community pharmacies. However, over half of the participants, 53.5% (n = 107) expressed doubts that private clinics would not like it, while 51% (n = 102) believed that it would require more time and effort to educate patients about the necessity of undergoing a test before taking antimalarial medicines.The mean score of the stated disadvantages varied between 1.22 (SD = 0.42) and 1.53 (SD = 0.50) (Table 7).

**Table 7:** Disadvantages of licensing use of m-RDT in community pharmacies

Items/statements	Disagree		Agree		Mean (SD)
	n	%	n	%	
People will not afford the cost of m-RDT and antimalarial in case of a positive test.	156	78.0	44	22.0	1.22 (0.42)
People will fear the finger prick necessary for needle injection	149	74.5	51	25.5	1.25 (0.44)
People will do self-treatments with antimalarial than never	140	70.0	60	30.0	1.30 (0.46)
People will tend to seek treatment in retail Pharmacies for even severe malaria	104	52.0	96	48.0	1.48 (0.50)
People will think Pharmacies want more money from the test	119	59.5	81	40.5	1.41 (0.49)
It will take more time to educate that a test is needed prior to antimalarial medicines	98	49.0	102	51.0	1.51 (0.50)
Regulatory authorities will not allow performing m-RDTs in Retail Pharmacies	106	53.0	94	47.0	1.47 (0.50)
Regulatory authorities will ask owners to hire lab technicians, thus additional cost.	127	63.5	73	36.5	1.37 (0.48)
Private health clinics will not like it	93	46.5	107	53.5	1.53 (0.50)
People will still ask for antimalarial medicines even when the test is negative.	109	54.5	91	45.5	1.46 (0.50)

n: Frequency, %: Percentage, SD: standard deviation

### Pharmacists’ views on what is needed to improve their contribution to malaria management

Community pharmacists have expressed their views on what is needed to increase their role in malaria management. These views were classified into the following five categories: [1] Licensing the use of malaria rapid diagnostic tests in Community Pharmacies, [2] improving the availability of malaria rapid diagnostic tests in community Pharmacies, [3] improving public-private sector collaboration, and [4] providing malaria training to community Pharmacists.

### Licensing the use of malaria rapid diagnostic tests in Community pharmacies

In Rwanda, the use of m-RDTs in community pharmacies remains unlicensed. However, community pharmacists have recommended that licensing their use could have several advantages. For instance, it could help prevent the presumptive treatment of malaria and promote evidence-based treatment, ultimately leading to a reduction in antimalarial drug resistance. Some of the participants explained that:

“Regulatory authorities should allow community pharmacists to perform rapid malaria diagnostic tests because many clients are seeking to buy antimalarial drugs in community pharmacies which may lead to the irrational use, hence antimalarial resistance “(Female Community Pharmacist, aged 28 years).

Although community pharmacists indicated a need to be allowed to perform malaria diagnostic tests. However, some also highlighted that severe malaria cases should be treated at the hospital. One participant stated:

“I think RDT can be legally used in community pharmacies. This will minimize the over-the-counter use of antimalarial medicines which is common in our daily practice. Though not allowed, it’s done by many pharmacies. This will minimize resistance, increase adherence, increase evidence-based treatment, and minimize treatment failures. But in case of severe malaria, pharmacists should immediately refer the patients to the hospital for further management (“Male community Pharmacist, aged 33 years).

Another one has put it:

“My view is that m-RDT should officially and legally be used in community pharmacies. However, we should reserve treatment of severe malaria to the hospital settings which have all the necessities” (male Community Pharmacist, aged 28 years).

### Improving the availability of malaria rapid diagnostic tests in community pharmacies

Even though m-RDTs are currently not licensed for use in community pharmacies, community pharmacists have suggested that making them more widely available could increase their usage. This, in turn, could lead to evidence-based malaria treatment, which would ultimately result in improved accessibility for the public and help prevent antimalarial drug resistance. One participant expressed that:

“Availability of rapid diagnostic tests could help a pharmacist to provide good pharmacy practice and evidence-based treatment hence reduction of antimalarial drug resistance and increased adherence by a patient as well as an increase in confidence to the pharmacist” (Male community Pharmacist, aged 25 years).

### Improving public-private sector collaboration

Community pharmacists have suggested that the national malaria program could collaborate with them to raise public awareness about the importance of dispensing antimalarial medicines only after a diagnostic test has been conducted. To facilitate this, pharmacists have proposed that the national malaria program and its partners could provide free malaria diagnostic tests to community pharmacies. This would help to improve the early detection of malaria at the community level. Some participants explained that:

“There is a need for collaboration between the public and private sector by sensitizing the public that a malaria test is important before asking for antimalarial medicines in community pharmacies” (Female Community Pharmacist, aged 29 years).

“Rwanda has a malaria program that offers malaria diagnostic tests to community health workers. I think it will be better if the government/partners also offer these tests to community pharmacies free of charge, then patients will only have to pay for the services of testing and the medication.” (Male community Pharmacist, aged 28 years).

“Community Pharmacy should be introduced in the national healthcare strategic plan and be given necessary training on malaria management as those from the public health sector.”(Male community Pharmacist, aged 30 years).

“If Community Health Workers can perform malaria tests and provide malaria treatment, community Pharmacists should also be trusted as healthcare professionals. It should be noted Pharmacists are more knowledgeable in mechanisms of medicines than any other healthcare professionals.” (Male Community Pharmacist, aged 34 years).

### Providing malaria training to community pharmacists

Community Pharmacists have acknowledged that they need training on malaria diagnostic tools. They have suggested to the Ministry of Health to organize training programs for community pharmacists to help them better manage malaria cases. They have also stressed that they are an underutilized resource in healthcare and could play a vital role in the proper management of malaria. By enhancing their knowledge and skills through training, community pharmacists could make even greater contribution to the fight against malaria and improve public health outcomes in their communities. Some participants stated that:

“Community Pharmacists are unutilized human resources in the management of malaria. The Ministry of Health should include them in training on such simple rapid tests including malaria.” (male community Pharmacist, aged 31 years).

“My view is that community pharmacists must be able to distinguish the severity of malaria, simple to severe,and know how to help, knowing that they cannot treat a severe form of malaria. Secondly, if possible be reminded about Malaria treatment protocols which are up to date. Those are my opinions because pharmacists are healthcare professionals with huge knowledge.”(Male community Pharmacist, aged 33 years).

“Where deemed necessary, training should be provided to community Pharmacists on effective use of RDTs. Therefore, new policies to train them must be put in place.” (Female Community Pharmacist, aged 30 years).

## Discussion

This study assessed the availability of m-RDTs in community pharmacies in Rwanda, determined the knowledge of community pharmacists on the use of m-RDTs, and explored community Pharmacists’ perceptions of the advantages and disadvantages of licensing their use in these settings. The study revealed that 27% of community pharmacies stocked m-RDTs, with availability being significantly lower in pharmacies located in provinces than in Kigali city, which is the most urbanized. This is consistent with a study conducted in six malaria-endemic countries in Africa,South East Asia, and South America,which also reported limited availability of m-RDTs in private health facilities, particularly in rural areas [24].However, while the current study observed a significantly high availability of m-RDTs in the capital city, it contrasts with a study conducted in Nigeria, which reported a significantly lower availability of m-RDTs in urban areas [25]. The statistics indicate that only 13.2% of the Rwandan population resides in Kigali city, with the majority residing in the provinces [26]. Thus, this suggests that expanding access to m-RDTs in the provinces is more critical.

Compared to similar studies in the region, the availability of m-RDTs in the current study is generally lower. For instance, in Uganda, m-RDTs were available in over half of registered pharmacies, while, in Kenya, one-third of registered pharmacies stocked m-RDTs [11, 27]. Rwanda’s current regulations permit community pharmacies to retail m-RDTs, but not to use them for malaria diagnostic testing purposes. This may be the major contributing factor to the limited availability of m-RDTs observed in the current study. Unfortunately, this limits the ability of community pharmacists to provide comprehensive care for malaria, highlighting the need for policy changes that could expand the scope of services offered by community pharmacies in Rwanda. Furthermore,the limited availability of m-RDTs hinders efforts to ensure widespread access to confirmatory testing [28]. This is particularly problematic given the crucial role that community pharmacies play in providing treatment for malaria.

In light of the critical role that m-RDTs play in detecting malaria parasites, community pharmacists must acquire a thorough grasp of their usage. This knowledge not only facilitates clear explanations for the public but also reinforces the role of community pharmacists as reliable sources of information regarding these diagnostic tests.Unfortunately, this study found limited knowledge among community Pharmacists on the use of m-RDTs. These results are at variance with a study from Nigeria where more than half of community Pharmacists had good knowledge of using m-RDTs to diagnose malaria [29]. Community Pharmacists in Rwanda can be comprehended from the aspect that regulatory authorities restrict the use of m-RDTs in community pharmacies. However, the significant association observed between the familiarity of community pharmacists with using m-RDTs and the availability of m-RDTs in their pharmacies suggests that additional training could be a valuable approach to enhance m-RDTs availability. In some countries like Nigeria, community Pharmacists are satisfactorily involved in malaria management [8].Interestingly, in the current study community pharmacists acknowledged that they have limited knowledge due to limited training. Thus ,they suggested additional training once m-RDTs are licensed for use in community pharmacies, as well as being integrated into the national malaria programs. In a previous study, community Pharmacists in Rwanda also demonstrated that lack of training was among the barriers limiting the provision of health promotion in their settings [30]. This finding is consistent with a study conducted in Nigeria [31].

Our study found that the majority, 91.5%, agreed that performing m-RDTs prior to dispensing antimalarial medicines contributes to a reduction of the use of antimalarials for other malaria-like symptoms. Additionally, 91.5% also expressed that the use of m-RDTs helps patients to find alternative treatment in case of a negative test, and that using m-RDTs promotes rational use of antimalarial medicines. These results are supported by previous studies in Kenya [9].In Nigeria ,a study also found that almost all the participants recognized the importance of confirming a malaria diagnosis prior to treatment [32].The views of most community pharmacists in the current study also expressed a willingness to contribute to malaria management. They reported that they are unutilized human resources who could help in malaria diagnosis and treatment, promote the rational use of malaria medicines, and help in the fight against the reported artemisinin resistance [16]. They also argued that their expertise should be trusted, and they are willing to collaborate with national malaria programs. As in the previous study [30], they suggested regulatory authorities license the use of malaria rapid diagnostic tests in community pharmacies, believing that doing so would promote the rational use of malaria medicines. It is important to note that Medical Doctors and prescribers are still deficient in Rwanda, thus expanding malaria diagnosis to community pharmacies can lessen long queues and waiting times in public health facilities. They suggested that a national malaria program could offer free tests to community pharmacies, allowing patients to pay only for the testing and medication. By expanding access to testing through community pharmacies, more people would have access to the care they need, ultimately reducing the burden of malaria in the community.

Given the importance of m-RDTs in expanding access to diagnostic testing for malaria, efforts should be made to reinforce factors that improve the availability of these tests. In the current study, several factors were found to be significantly associated with the availability of m-RDTs including the location of the pharmacy, the presence of nurses in the pharmacy, requests to sell m-RDTs, and familiarity with using m-RDTs. This finding suggests that the demand for m-RDTs among clients seeking antimalarials can drive the availability of these tests. It also suggests that collaboration between community pharmacists and nurses could improve the availability of m-RDTs and increase the uptake of diagnostic testing for malaria. Furthermore, it highlights the importance of training and education programs for healthcare providers to increase their knowledge and skills in using m-RDTs. Close to our study, a study in Ghana found that the presence of m-RDTs cut the likelihood of selling antimalarial medications without a test by 42% [33]. These findings suggest that a multi-faceted approach may be needed to address barriers to the availability of m-RDTs in community pharmacies in Rwanda, which could include promoting the use of m-RDTs in cases where antimalarials are requested, providing training and support for healthcare workers, and developing targeted interventions to increase the availability of m-RDTs in rural areas. This study, therefore, highlights the importance of considering contextual factors when developing interventions to enhance access to m-RDTs through community pharmacies. Such programs could be instrumental in increasing the availability of m-RDTs and improving the quality of malaria diagnosis and management.

The present study had strengths. First of all, this study was important because no other studies had been conducted among community pharmacists to understand their perspectives on what advantages or disadvantages can be of using m-RDTs in community pharmacies. Secondly, this study used a validated instrument for data collection, which indicated a satisfactory consistency (Cronbach’s Alpha, α = 0.89). Thirdly, the conclusions of this study are valid and have a national representation because it was carried out nationally utilizing a variety of methods. However, some limitations warrant discussions. The study did not consider the perspectives of regulatory authorities regarding licensing the use of m-RDTs in community pharmacies. Another limitation was sampling method. It’s important to acknowledge that there may be some differences in the characteristics or perspectives of participants who are selected using a random sampling method versus a purposive sampling method. Therefore, our sampling method might have led to some bias and thus, future research using a different sampling method will be necessary.

## Conclusions

The current study investigated the availability of m-RDTs, assessed the knowledge of community pharmacists on the use of m-RDTs, and explored Pharmacists’ perceptions of the advantages and disadvantages of licensing the use of m-RDTs. The findings indicated a low availability of m-RDTs and limited knowledge on use of m-RDTs among community pharmacists. However, community pharmacists showed positive attitudes towards dispensing antimalarial medicines based on a diagnostic test and are eager to contribute to rational malaria management. However, some barriers need to be addressed, such as revising the structure of the provision of healthcare services in community pharmacies, which restricts the use of malaria rapid diagnostic tests. Deregulating malaria rapid diagnostic tests and allowing their use in community Pharmacies can increase their availability, resulting in a reduction of overuse of antimalarial medicines. Therefore, Rwanda’s healthcare system and partners are recommended to design appropriate programs aiming to improve the involvement of community pharmacists in the management of malaria. The public health authorities in Rwanda can also take steps to disseminate knowledge on the use of malaria RDTs in community pharmacies. To achieve universal access to malaria diagnosis and treatment, there should be measures to close the testing ability and availability of m-RDTs Community Pharmacies. Thus, licensing the use of m-RDTs is believed to be the first step toward promoting the rational use of antimalarials in Rwanda.

## Data Availability

All relevant data are included in this manuscript. Data may be shared upon a reasonable request ,and is provided to the corresponding author.

## Abbreviations

WHO: World Health Organization
m-RDTs: Malaria rapid diagnostic tests
ACT: Artemisinin-based combination therapy
NPC: National Pharmacy Council
IRB/CMHS: Institutional Review Board, College of Medicine, and Health Sciences
SSA: Sub-Saharan Africa
